# The Cervico-Parasternal Thoracotomy (CPT): A New Surgical Approach for the Resection of Cervicothoracic Neuroblastomas

**DOI:** 10.3390/children8030229

**Published:** 2021-03-17

**Authors:** Giuseppe Martucciello, Federica Fati, Stefano Avanzini, Filippo Maria Senes, Irene Paraboschi

**Affiliations:** 1 Dipartimento di Neuroscienze, Riabilitazione, Oftalmologia, Genetica e Scienze Materno-Infantili (DiNOGMI), University of Genoa, 16147 Genoa, Italy; fedes.fati@gmail.com (F.F.); i.paraboschi@ucl.ac.uk (I.P.); 2 Department of Paediatric Surgery, IRCCS Giannina Gaslini Children’s Hospital, 16147 Genoa, Italy; stefanoavanzini@gaslini.org; 3 Reconstructive Surgery and Hand Surgery Unit, IRCCS Giannina Gaslini Children’s Hospital, 16147 Genoa, Italy; filipposenes@gaslini.org

**Keywords:** neuroblastoma, cervical tumours, mediastinal tumours, thoracotomy

## Abstract

Cervicothoracic neuroblastomas (NBs) pose unique surgical challenges due to the complexity of the neurovascular structures located in the thoracic inlet. To date, two main techniques have been reported to completely remove these tumours in children: the trans-manubrial and the trap-door approaches. Herein, the authors propose a third new surgical approach that allows a complete exposure of the posterior costovertebral space starting from the retro-clavicular space: Cervico-Parasternal Thoracotomy (CPT). The incision is made along the anterior margin of the sternocleidomastoid muscle until its sternal insertion, and then the incision proceeds vertically following the ipsilateral parasternal line. The major pectoralis muscle is detached, and the clavicle and the ribs are disarticulated from their sternal insertions. Following an accurate isolation of the major subclavian blood vessels and the brachial plexus roots, the tumour is then completely exposed and resected by switching from a frontal to a lateral view of the costo-vertebral space. By adopting this technique, five cervicothoracic NBs were completely resected in a median operative time of 370 min (range: 230–480 min). By proceeding in safety with the heart apart, neither vascular injuries nor nerve damages occurred, and all patients were safely discharged in a median postoperative time of 11 days (range: 7–14 days). At the last follow-up visit (median: 16 months, range: 13–21 months), all patients were alive and disease-free.

## 1. Introduction

Surgery represents a cornerstone within the extensive multimodal treatment of neuroblastoma (NB). The goal of surgery is to achieve a complete tumour excision preserving the function of the surrounding healthy parenchyma and the frequently involved neurovascular structures.

The surgical resection of cervicothoracic NBs is a technically challenging procedure made particularly demanding by the common encasement of the subclavian vessels and brachial plexus and by the extensive fibrosis resulting from the neoadjuvant chemotherapy.

Although the prognosis of these tumours is usually good thanks to the high prevalence of localized forms with favourable MYCN status, intra-operative complications have been frequently reported [1].

The standard surgical approaches, such as a simple cervicotomy, thoracotomy or combined techniques, have been abandoned because they were unable to provide an adequate control of the complex neurovascular structures located in the thoracic inlet.

Therefore, more recently, two main procedures have been introduced to allow a proper exposure of the surgical field: the trans-manubrial and the trap-door approaches [2,3,4,5,6,7,8,9,10,11].

**Figure 1:**
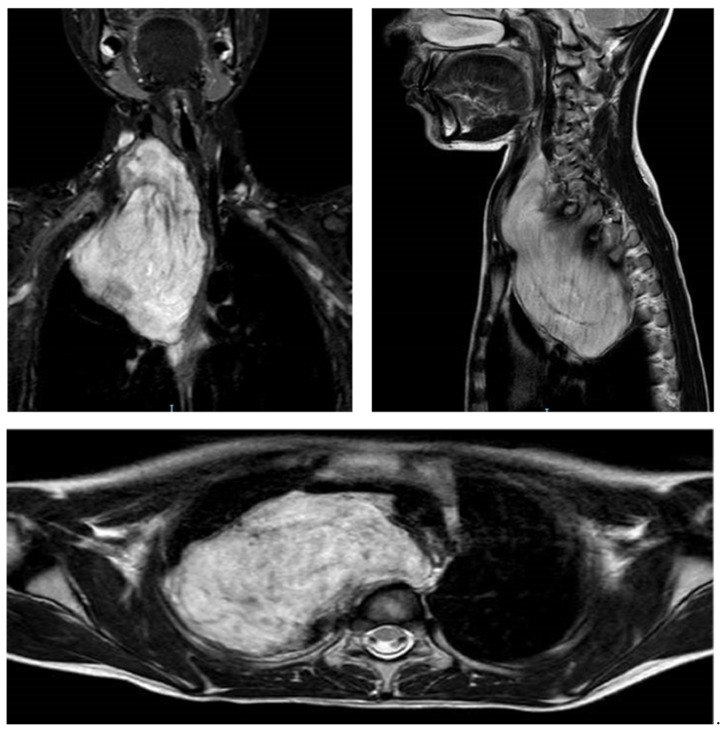
MRI scans of a patient affected by a cervicothoracic NB treated with CPT type B. The tumour causes the antero-lateral dislocation of the internal jugular vein, the right carotid artery, the trachea, and the bronchial biforcation. Posteriorly it penetrates into the epidural space (T1–T3), without compressing it. Inferiorly it extends toward the interscalen space along the brachial plexus. The subclavian and vertebral artery are in strict contact but not infiltrated by the tumour.

Herein, the authors reported a new third surgical technique (based on the surgical experience of F.M.S. and G.M.), which allows not only an adequate control of the vital structures located in the thoracic inlet, but also an excellent exposure of the posterior costal–vertebral space required to obtain a complete resection of the extraspinal components of the tumour: Cervico-Parasternal Thoracotomy (CPT) (Figure 1.).

## 2. Description of the Operative Technique

### 2.1. Patient Positioning

The patient is placed supine with the head extended and rotated contralaterally to the tumour.

### 2.2. Surgical Incision

**Figure 2:**
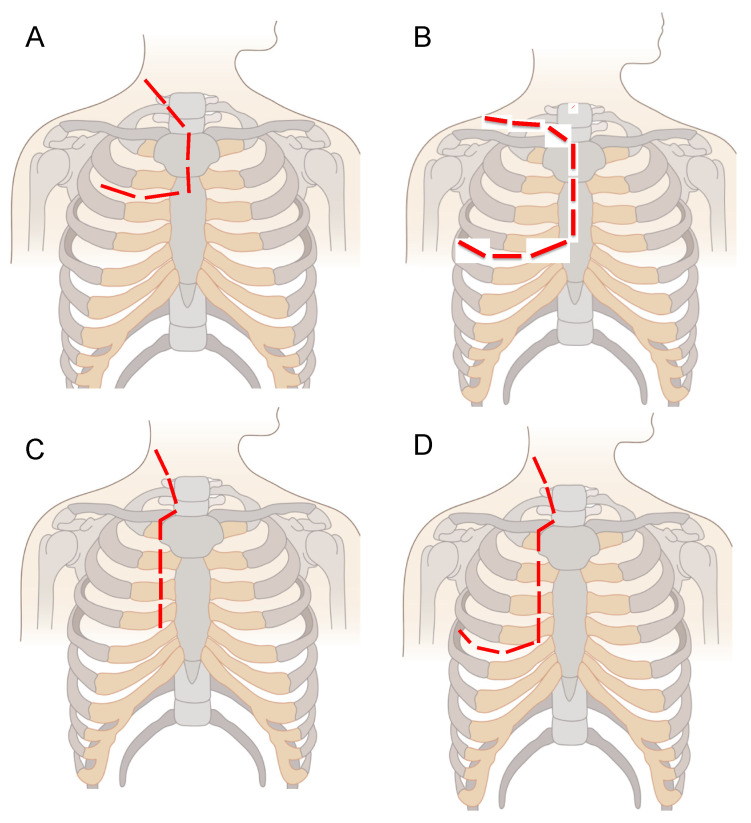
Main surgical approaches for the resection of cervicothoracic NBs. The red line shows the site of the surgical incision. (**A**). The Trans-Manubrial Sternotomy (TMS). (**B**). The Trap-Door Approach (TDA). (**C**). Cervico-Parasternal Thoracotomy (CPT) type A. (**D**). Cervico-Parasternal Thoracotomy (CPT) type B.

**Figure 3:**
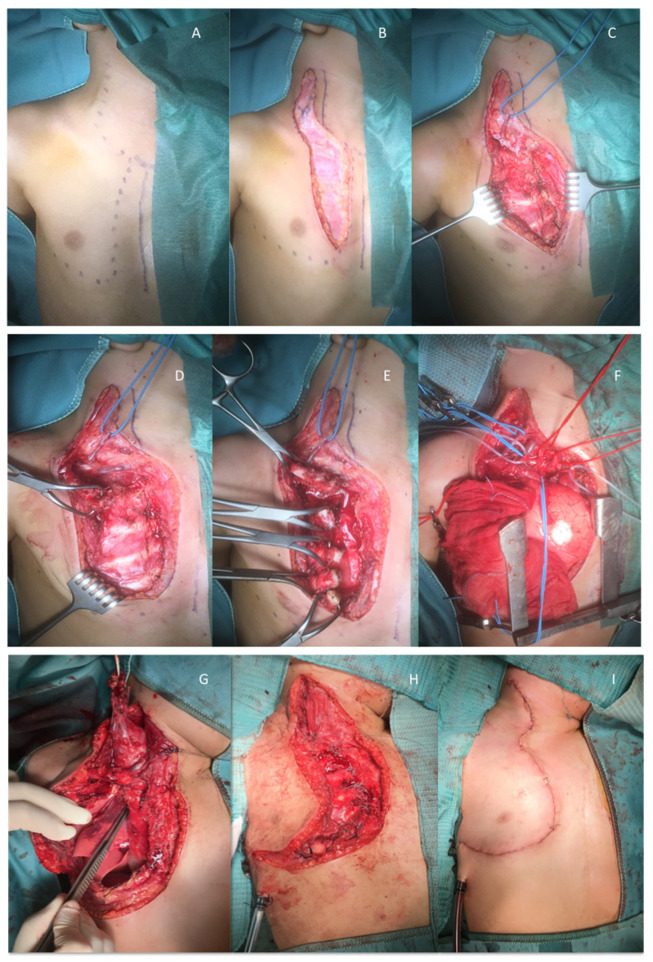
Main steps of the Cervico-Parasternal Thoracotomy (CPT). (**A**). Incision line for CPT type A. (**B**,**C**). Surgical dissection of the subcutaneous tissue. (**D**,**E**). Detachment of the major pectoralis muscle and identification and disarticulation of the clavicle and the ribs from their sternal insertion. (**F**). Identification and isolation of vital structures: common carotid artery (red elastic wire), subclavian artery (red elastic wire), internal jugular vein (blue elastic wire), phrenic nerve (white elastic wire). (**G**–**I**). Main steps and incision line for CPT type B.

The incision is made following the anterior margin of the sternocleidomastoid muscle down to the sterno-clavicular junction. In order to perform CPT type A, the incision line proceeds vertically down along the right parasternal line (Figure 2C and Figure 3A–C). However, in selected patients when the tumour is more widely extended, CPT type B is the preferred surgical approach (Figure 2D and Figure 3G–I). In these cases, the incision is extended laterally to perform an anterior-lateral thoracotomy, providing a postero-lateral or “door” access.

### 2.3. Thoracic Access

The subcutaneous tissue is dissected, the muscular fascia is identified, and the major pectoralis muscle is detached from its sternal insertions (Figure 3D). The clavicle and the ribs are disarticulated at the level of their sternal insertions by using electrocautery devices and the LigaSure™ sealer (Medtronic, Minneapolis, MN, USA) (Figure 3E). The articular structures at their joint junctions are preserved in order to allow a complete reconstruction at the end of the surgical procedure. In relation to the mediastinal extension of the tumour, a thoracotomy at the level of the 4th and 5th rib may be required to permit a more radical excision.

### 2.4. Tumour Resection

The main blood vessels (corresponding to the internal jugular vein the subclavian artery, the common carotid artery, and the brachiocephalic artery) are identified and carefully isolated (Figure 3F). An electrical nerve stimulation is performed to identify and preserve the brachial plexus. The tumour is detached and completely excised by using the LigaSure™ sealer (Medtronic, Minneapolis, MN, USA).

### 2.5. Surgical Reconstruction and Closure 

At the end of the surgical procedure, a chest drain is placed in situ, and the ribs are sutured at their sternal insertions by using non-absorbable stiches (Ti-cron TM 2/0, Medtronic, Minneapolis, MN, USA). Muscle and subcutaneous layers are repaired in an anatomical fashion by using absorbable sutures. The skin is closed with an absorbable subcuticular suture, and adhesive strips are placed to protect the surgical wound (Figure 3H,I).

## 3. Results

During the study period (January 2017–December 2019), five patients (2 females; 3 males) affected by cervicothoracic NBs were treated by using the CPT approach.

Patient demographic and tumour findings are reported in Table 1.

The CPT provided an optimal exposure of the surgical field by switching from a frontal to a lateral view of the costo-vertebral space. By working in safety with more operative space available, a gross total resection was achieved in all patients in a median operative time of 370 min (range: 230–480 min).

Due to the wide and all-round view of the tumour, no intra-operative vascular injuries occurred. A single patient had a foraminal residue, which was considered acceptable and did not worsen the prognosis.

The postoperative course was well tolerated in all patients with the aid of the combination of opioid analgesics, non-steroidal anti-inflammatory drugs, and paracetamol.

A prolonged analgesia was not required in our patients that were discharged from the ICU in a median postoperative time of 46 h (range: 24–48 h).

In the postoperative follow-up, no post-operative complications occurred, and all patients were discharged in a median postoperative time of 11 days (range: 7–14 days).

In particular, no neurological deficits were recorded in the median follow-up period of 16 months (range: 13–21 months). One single patient displayed persisting symptoms of the Bernard-Horner (BH) syndrome, which were present even before surgery. No pulmonary disorders, orthopaedic or spinal deformities were registered. However, all patients wore an orthopaedic corset for the first two postoperative months to prevent chest wall instabilities or thoracic asymmetries. The use of the corset should just to be considered a safety precaution the authors’ adopted for treating the first five cases. The acquisition of more experience with this approach will define if it should be considered mandatory during the postoperative care of these children. All of them had complete stabilization of the shoulder joint and no cervical defects were reported.

The surgical resection and the postoperative management proved to be effective, and all five patients were alive and disease-free at the last follow-up.

## 4. Discussion

Cervicothoracic NBs, arising from the paravertebral cervicothoracic ganglia, account for fewer than 5% of cervicothoracic tumours [11]. As in other locations, the prognosis of localized NBs is usually good and mainly depends on a complete tumour excision [12]. However, the surgical resection of cervicothoracic tumours is a technically demanding procedure due to the limited access to this unique anatomical region and the frequent encasement of the neurovascular structures located there.

To date, two main surgical techniques have been popularized for the excision of this subgroup of NBs: the trans-manubrial and the trap-door approaches [2,3,4,5,6,7,8,9,10,11,12].

The trans-manubrial technique involves an L-shaped skin incision which extends from the level of the thyroid cartilage downward along the anterior margin of the sternocleidomastoid muscle onto the manubrium and the upper sternum. It is then prolonged in an anterior thoracotomy at the level of the second rib. By using the trans-manubrial access, the clavicle is not dissected from the sternum; therefore, a complete view of retro–clavicular and sub-clavicular space is not always guaranteed. This approach affords the access to the subclavian region with a good control of the vascular structures (carotid, subclavian and vertebral arteries, subclavian and jugular veins) and nerves (brachial plexus, phrenic nerve, vagus nerve, recurrent laryngeal nerve) which pass through this anatomical region; however, the risk of complications cannot be excluded [3,4,8,10,11] (Table 2). Moreover, the trans-manubrial approach is not to be considered the first-line treatment in the case of cervicothoracic tumours extending deeply into the posterior mediastinum.

On the other hand, the trap-door approach begins with a supraclavicular incision, descending to the mid-portion of the sternum down to third or fourth intercostal space and then going laterally through the corresponding interspace to the mid-axillary line. By involving a partial sternotomy combined with a traditional thoracotomy, the trap-door approach creates a rib-flap that can be retracted laterally in children. By using this approach, better access to the sub-clavicular and thoracic spaces is provided compared to the trans-manubrial technique.

Furthermore, by splitting the sternum, the trap-door approach allows good access to the thoracic cavity thanks to the huge flexibility of the child bony cage. However, this predominantly results in a frontal vision of the surgical field.

However, by adopting this technique, there is also the burden of a high risk of surgical complications that have been reported Amongst them, diaphragmatic paresis and Bernard-Horner Syndrome are the most frequently reported [2,5,6] (Table 2).

Herein, the authors reported an alternative technique of these two surgical approaches to provide a prevalent postero-lateral exposure of the costal–vertebral space: cervico-parasternal thoracotomy (CPT).

In the reported experience, the procedure proved to be safe and well tolerated. It allowed us to accomplish the main goal of achieving a complete excision of the tumour and of its extraspinal components.

No intra- or post- operative complications were recorded in a surgical procedure associated with excellent results. In particular, in the five operated cases, no haemothorax, pleural effusion, chylothorax, gastroparesis, swallowing difficulty, or diaphragmatic paralysis was reported. Moreover, the final cosmetic aspect was excellent, and no chest-wall deformities occurred thanks to the accurate preservation of the osteomuscular structures responsible for the elasticity of the bony cage. The persistent Bernard-Horner Syndrome affecting one single patient even before surgery should be considered an almost unavoidable dysfunction caused by the tumour involvement of the stellate ganglion.

Even if this innovative approach cannot be considered completely free from complications, the authors believe that the parasternal retraction, as opposed to the sternotomy, allows good access to the sub-clavicular spaces and to the posterior mediastinum. The excellent exposure of the posterior costal-vertebral space, the area where the tumours usually originate, facilitates their radical resection thanks to the optimal control of the vascular, lymphatic, and nervous structures involved.

The CPT technique respected all the oncological criteria and permitted an adequate and safe surgical resection, without major intraoperative risks.

The limitations of this study include all the issues related to the retrospective analysis and the small number of patients involved. Moreover, a longer follow-up period is required to evaluate late-occurring complications and cosmetic results over time. All these elements prevent the authors from drawing any significant comparison between the three surgical approaches, which have been all successfully applied to the surgical treatment of cervicothoracic NBs.

The main value of this paper relies on describing a new surgical approach which has proved to be useful in achieving good exposure of the thoracic inlet and the posterior costo-vertebral space with minimal postoperative morbidities. Moreover, a brief literature review of the main surgical approaches applied for the surgical treatment of this childhood tumour has been presented to provide a complete spectrum of the surgical alternatives available.

Considering the rarity of NB tumours at this site, we recommend this technique as it has proved to be useful in achieving a good exposure of the thoracic inlet and the posterior costo-vertebral space with minimal postoperative morbidities.

## Figures and Tables

**Table 1:** Patient demographic and tumour findings.

	PATIENT 1	PATIENT 2	PATIENT 3	PATIENT 4	PATIENT 5
Age at surgery (years)	11	9	2	2	7
Gender	male	female	female	male	male
Site	right	right	right	right	right
ASA status	2	3	3	3	3
IN Staging System	III	III	III	III	IV
INRG Staging System	L2	L2	L2	L2	M
MYCN status	not amplified	not amplified	not amplified	not amplified	amplified
Histology	favourable	favourable	favourable	favourable	unfavourable
Previous surgery	no	yes (laminectomy D5–D12)	no	no	yes (recurrent NB)
Operative time (min)	240	480	480	370	230
RBC transfusion	no	yes	yes	no	yes
Hospital stay (days)	10	13	14	11	7
ICU stay (hours)	24	48	72	96	24
Preoperative chemotherapy	no	yes	yes	yes	yes
Postoperative therapy	no	no	cis-retinoic acid)	radiotherapy	cis-retinoic acid & immunotherapy comprising anti-GD2 antibody (ch14.18) and IL-2
Postoperative complications	no	no	no	no	no
Follow up (months)	21	18	13	15	17
Follow up status	alive, disease-free	alive, disease-free	alive, disease-free	alive, disease-free	alive, disease-free

**Table 2:** Main articles reporting the surgical experience with cervicothoracic neuroblastomas in children.

AUTHORS	PATIENTS’ POPULATION	AGE (Average, Range; Months)	SURGICAL APPROACH	OPERATIVE TIME (Average, Range; Minutes)	TUMOUR TYPE	HOSPITAL STAY (Average, Range; Days)	COMPLICATIONS	SURVIVAL OUTCOMES	FOLLOW-UP PERIOD (Average, Range; Months)
Pranikoff T et al., 1995 [9]	2	10.5 (4–17)	Trap-door (*n* = 2)	nd	Ganglioneuroblastoma (*n* = 2)	7.5 (5–10)	None	nd	nd
Sauvat F et al., 2006 [11]	4	35 (10–84)	Trans-manubrial (*n* = 4)	nd	Neuroblastoma (*n* = 4)	nd	Chylotorax (*n* = 1); gastroparesis (*n* = 1); diaphragmatic paresis (*n* = 2); Bernard–Horner Syndrome (*n* = 4)	Alive: 4/4 (100.0%); complete remission: 4/4 (100.0%)	nd (8–32)
Pimpalwar AP et al., 2007 [8]	1	24 (na)	Trans-manubrial (*n* = 1)	160	Ganglioneuroblastoma (*n* = 1)	2	Bernard–Horner Syndrome (*n* = 1)	Alive: 1/1 (100.0%); complete remission: 1/1 (100.0%)	6 (na)
Jones vs. et al., 2008 [5]	1	42 (na)	Trap-door (*n* = 1)	nd	Ganglioneuroma (*n* = 1)	nd	Bernard–Horner Syndrome (*n* = 1)	Alive: 1/1 (100.0%); complete remission: 1/1 (100.0%)	3 (na)
Parikh D et al., 2011 [7]	3	24 (nd)	Trap-door (*n* = 3)	nd	Neuroblastoma (*n* = 4)	nd	nd	Alive: 2/3 (66.7%); complete remission: 2/3 (66.7%)	57.3 (16–96)
De Corti et al., 2012 [3]	8	45.6 (nd)	Trans-manubrial (*n* = 6); trap-door (*n* = 2)	263.8 (140–410)	Neuroblastoma (*n* = 5); ganglioneuroblastoma (*n* = 3)	11.9 (9–26)	Chylotorax (*n* = 1); hemothorax (*n* = 1); Bernard–Horner Syndrome (*n* = 3)	Alive: 8/8 (100.0%); complete remission: 7/8 (87.5%)	24
McMahon et al., 2013 [6]	1	48 (na)	Trap-door (*n* = 1)	nd	Ganglioneuroblastoma (*n* = 1)	nd	Bernard–Horner Syndrome	Alive: 1/1 (100.0%); complete remission: 1/1 (100.0%)	60
Qureshi SS et al., 2014 [10]	7	36 (11–72)	Trans-manubrial (*n* = 4); trap-door (*n* = 3)	327 (69–240)	Neuroblastoma (*n* = 7)	6.5 (5–10)	Diaphragmatic paralysis (*n* = 1)	Alive: 5/7 (66.7%); complete remission: 5/7 (66.7%)	nd
El Madi A et al., 2007 [4]	9	71.8 (4–188)	Trans-manubrial (*n* = 9)	nd	Neuroblastoma (*n* = 6); ganglioneuroblastoma (*n* = 3)	7	Chylothorax (*n* = 2); Bernard–Horner Syndrome (*n* = 2); diaphragmatic paralysis (*n* = 2); left hand paresthesia (*n* = 1)	Alive: 7/9 (77.8%); complete remission: 4/9 (44.4%)	92.6 (3–190)
Chui CH et al., 2020 [2]	21	42 (3.6–94.8)	Trap-door (*n* = 21)	312 (150–546)	Neuroblastoma (*n* = 18); ganglioneuroblastoma (*n* = 3)	nd	Klumpke’s palsy (*n* = 1); winged scapula (*n* = 1); diaphragmatic paralysis (*n* = 1); bronchomalacia (*n* = 2); Bernard–Horner Syndrome (*n* = 21)	Alive: 16/21 (75.0%); complete remission: nd	33.6 (3.6–92.4)

Abbr. na: not applicable, nd: not determined.

## Data Availability

All these data have been collected and registered on an electronic database (Microsoft Excel 2007, Redmond, WA, USA) kept in IGG secure computer. The archiving length will be 25 years. Space for archiving hard copy files has been sufficient. No external archiving unit has been required. Professor G. Martucciello is responsible for data collection, recording and quality.
